# Research Progress of Photo-/Electro-Driven Thermochromic Smart Windows

**DOI:** 10.3390/nano11123335

**Published:** 2021-12-08

**Authors:** Xiaotong Zou, Haining Ji, Yong Zhao, Mingying Lu, Jundong Tao, Pinghua Tang, Bin Liu, Xitao Yu, Yuliang Mao

**Affiliations:** School of Physics and Optoelectronics, Xiangtan University, Xiangtan 411105, China; xiaotongzou_0705@163.com (X.Z.); yongzhao_0912@163.com (Y.Z.); 202021001488@smail.xtu.edu.cn (M.L.); JundongTao_314@163.com (J.T.); pinghuatang@xtu.edu.cn (P.T.); bl987@uowmail.edu.au (B.L.); yxt1240710760@163.com (X.Y.)

**Keywords:** photo-/electro-driven, thermochromic, smart windows, research progress

## Abstract

Thermochromic smart windows can automatically control solar radiation according to the ambient temperature. Compared with photochromic and electrochromic smart windows, they have a stronger applicability and lower energy consumption, and have a wide range of application prospects in the field of building energy efficiency. At present, aiming at the challenge of the high transition temperature of thermochromic smart windows, a large amount of innovative research has been carried out via the principle that thermochromic materials can be driven to change their optical performance by photothermal or electrothermal effects at room temperature. Based on this, the research progress of photo- and electro-driven thermochromic smart windows is summarized from VO_2_-based composites, hydrogels and liquid crystals, and it is pointed out that there are two main development trends of photo-/electro-driven thermochromic smart windows. One is exploring the diversified combination methods of photothermal materials and thermochromic materials, and the other is developing low-cost large-area heating electrodes.

## 1. Introduction

Rapid increasing energy consumption leads to an energy shortage, accompanied by environmental pollution. In developed countries, building energy consumption accounts for 20–40% of the total energy consumption [1]. Nowadays, some new technologies have been developed to adjust the indoor temperature and reduce building energy consumption, such as smart windows. In 1985, M. Lampert and C. G. Granqvist et al. [2] first proposed electrochromic materials for smart windows. Smart windows are composed of glass or transparent materials as substrates and dimming materials. Under certain conditions, the transparency can be adjusted to regulate the amount of sunlight, which can effectively save energy. According to different excitation means, smart windows can be divided into electrochromic, photochromic and thermochromic smart windows. Thermochromic smart windows have been widely investigated by researchers due to their simple structure, low preparation cost and active response to external temperature stimulation. However, when the external temperature is lower than the switching threshold, the optical performance of the thermochromic smart window is difficult to change. Therefore, many experts and scholars have combined photothermal or electric heating control to assist in driving the thermochromic smart window to achieve the transformation of optical performance at room temperature, further enhancing the applicability of the thermochromic smart windows.

At present, photo-/electro-driven thermochromic smart windows are mainly divided into VO_2_-based composites, hydrogels and liquid crystals.

VO_2_ is the most widely used inorganic thermochromic material, and is used for its metal-to-insulator transition in smart windows, which is accompanied by significant changes in its electrical, optical and magnetic properties. This transition is due to a structural change from a monoclinic semiconductor phase to a metallic tetragonal rutile structure when the sample temperature is above 68 °C. Based on its excellent metal–insulator transition (MIT) properties, VO_2_ has attracted widespread attention from researchers in the field of smart windows [3,4,5,6] and infrared camouflage stealth [7,8,9,10,11,12]. However, in practical applications, VO_2_ still has challenges, such as its high phase transition temperature and poor optical performances.

Hydrogels with a low critical temperature have a wide range of tunable chemical and physical properties, making them ideal for use in smart windows. Hydrogels can reversibly shift between transparent/opaque states through hydrophilic/hydrophobic phase transitions, responding to changes in the ambient temperature. At low temperatures, the hydrogels show a transparent state due to the formation of hydrogen bonds between hydrophilic groups and water molecules. When the temperature rises to a critical temperature, the hydrophobic group leads to an opaque state due to hydrogen bond breakage. Compared with VO_2_, hydrogels are easy to prepare and have a low critical temperature. The lower critical solution temperature (LCST) of PNIPAm is only 32 °C. Therefore, they are also widely used in the field of smart windows [13,14].

However, the visible light transmittance of hydrogels at a high temperature is low, so it is necessary to adjust its thickness and design a suitable glass panel to increase the transparency of hydrogels before it can be used in the field of smart windows [15].

Similar to hydrogels, liquid crystals are mainly controlled by visible light. In thermodynamics, liquid crystals are between the crystalline solid state and the isotropic liquid state, and can simultaneously show the anisotropy of the crystals and the flow characteristics of the liquids. Thermochromic liquid crystals can change the arrangement and orientation of anisotropic molecules in response to the stimulation of temperature and voltage at the same time. Liquid crystal/polymer materials have multiple responsiveness and good mechanical properties, which make them widely used in smart windows [16].

**Figure 1:**
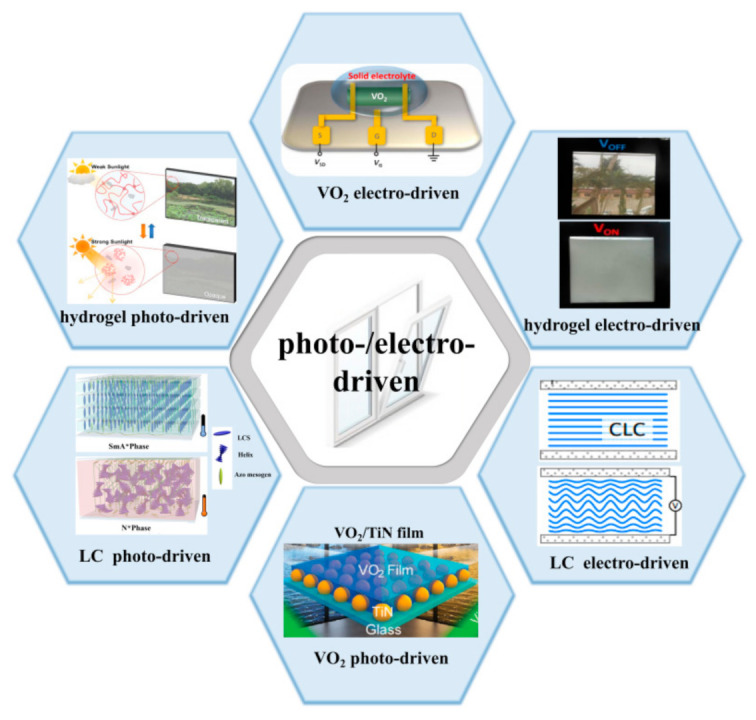
Types and working principle of photo- and electro-driven thermochromic smart windows.

Based on this, the paper mainly reviews photo- and electro-driven thermochromic smart windows, from the three aspects of VO_2_, hydrogels and liquid crystals, as shown in Figure 1.

## 2. Photo-Driven Thermochromic Smart Windows

The photo-driven thermochromic smart windows can actively adjust its optical properties in response to the changes in light radiation intensity and temperature, which is a completely passive way of light modulation. Compared with the single response of the traditional thermochromic smart windows, the photo-driven thermochromic smart windows realize the dual response of light and heat. It can respond to stimulation in areas with strong light radiation and a low temperature, and can regulate the sunlight, which further expands the application range of thermochromic smart windows.

### 2.1. VO_2_-Based Smart Windows

**Figure 2:**
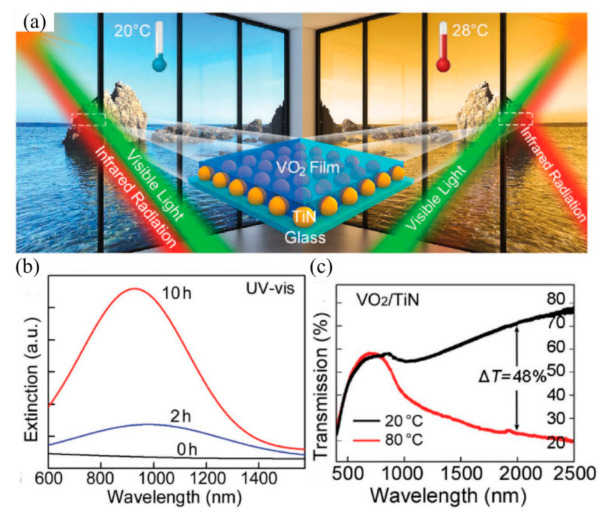
(**a**) Schematic diagram of VO_2_/TiN smart windows. (**b**) UV-Vis spectra of TiO_x_ (0 h), TiO_x_N_y_ (2 h), TiN (10 h) nanoarrays on quartz substrate. (**c**) Transmission spectra of VO_2_/TiN coatings at 20 °C and 80 °C. Reproduced with permission from [21]. Copyright 2018, Wiley.

In order to be close to practical applications, researchers usually reduce the phase transition temperature of VO_2_ via doping elements, but this will also lead to a decrease in its optical properties [17,18,19]. However, by combining VO_2_ with photothermal materials and using photothermal conversion, the sunlight is converted into heat energy, and VO_2_ can be driven to change the optical performance at room temperature. Ji et al. [20] designed and assembled a composite film of PbS and VO_2_. The UV-Vis-NIR light can be absorbed through the interaction of solar photons and PbS phonons and converted to heat energy, thus allowing for VO_2_ to undergo a phase change at room temperature. Subsequently, in order to further improve the optical performance of the photo-driven VO_2_ film, Hao et al. [21] prepared VO_2_/TiN smart coatings for room temperature applications by hybridizing thermochromic VO_2_ with plasmonic TiN nanoparticles (Figure 2a). The VO_2_ phase transition was accelerated by the strong plasma absorption in the near-infrared region of the TiN plasmonic nanoarray (Figure 2b). In addition, the VO_2_/TiN coating had a visible light transmittance of approximately 50% and an infrared conversion efficiency of 48% at 2000 nm, which effectively improved the optical performance of the light-driven VO_2_ composite films (Figure 2c).

### 2.2. Hydrogel Smart Windows

Hydrogels are popular materials for photo-driven thermochromic smart windows. The combination of hydrogels with graphene oxide (GO) [22,23,24,25], antimony-doped tin oxide (ATO) [26,27,28], Cs_x_WO_3_ [29] and other high-absorption materials can stimulate the transformation of the optical properties of the hydrogels, thereby improving the conversion rate of the hydrogels.

**Figure 3:**
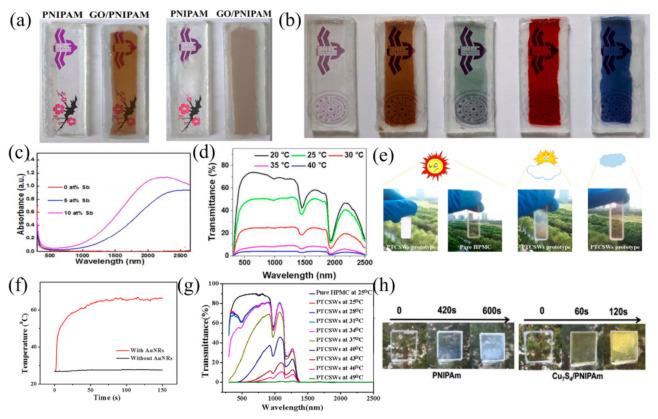
(**a**) Photos of the transparency/opacity transition of PNIPAm glass and GO/PNIPAm glass before and after 5 min of sunlight. Reproduced with permission from [25]. Copyright 2017, Elsevier. (**b**) Color GO/PNIPAm glass. Reproduced with permission from [25]. Copyright 2017, Elsevier. (**c**) Ultraviolet–visible–near infrared absorption spectra of 0.025 wt% SnO_2_ with Sb doping content of 0, 5 and 10%. Reproduced with permission from [27]. Copyright 2017, American Chemical Society. (**d**) Transmission spectra of 5HATO films at different temperatures. Reproduced with permission from [28]. Copyright 2018, Elsevier. (**e**) PTCSWs prototype and pure HPMC in the hot summer (left), digital photos of soft sunny summer days (middle) and cloudy days (right). Reproduced with permission from [31]. Copyright 2018, Elsevier. (**f**) Temperature rise trajectory of PVA/dye film with or without AuNRs. Reproduced with permission from [31]. Copyright 2018, Elsevier. (**g**) UV-Vis-NIR spectra of PTCSWs prototype. Reproduced with permission from [31]. Copyright 2018, Elsevier. (**h**) Photos of Cu_7_S_4_/PNIPAm and PNIPAm hydrogels under real sunlight in summer (31 °C). Reproduced with permission from [32]. Copyright 2019, Elsevier.

GO is a common photothermal material. Under medium-intensity visible light irradiation, the fully reversible volume phase transition of the hydrogels can be triggered by the photothermal effect of GO [22]. Kim et al. [22] first studied the optical properties of hydrogels driven by the heat generated via GO. Thus, we can develop the switchable glazing of a novel photothermotropic mechanism that screens strong sunlight and heat radiation in response to the sunlight intensity, as well as the temperature. Subsequently, Kim et al. [23] prepared a photo-driven thermochromic smart window by using GO and poly(N-isopropylacrylamide) (PNIPAm). Lee et al. [24] prepared a gradient copolymer hydrogel containing GO by manipulating the monomer composition to control the hydrophilic–hydrophobic balance of the copolymer. Copolymer hydrogels exhibit different thermal behaviors according to the monomer composition. With changes in the temperature and light radiation, copolymer hydrogels can be gradually dehydrated, resulting in almost linear transmittance changes. Besides, in order to create a more comfortable and colorful life, the color change in thermochromic smart windows, such as the warm/cool-tone, is also the focus of research. As can be seen from Figure 3a,b, GO embedded within the thermotropic hydrogels can absorb the colored organic solvent, can prepare the smart glasses with arbitrary color and can further promote the development of color-tuning smart windows [25].

When choosing photothermal materials, it is necessary to consider its influence on the visible light transmittance of windows and to avoid materials that act on the visible light region. ATO mainly regulates near-infrared light. Compared with PNIPAm/GO film, PNIPAm/ATO film has better solar modulation in the near-infrared region [26]. Besides, the effect of Sb doping in ATO on its photothermal properties was also studied. The results showed that the PNIPAm/ATO film with 10% Sb doping had the best response speed and solar modulation ability [26,27] (Figure 3c). With the increase in ATO content and film thickness, the solar modulation ability and response speed will also increase [28]. Subsequently, a supramolecular nanocomposite hydrogel film was prepared by integrating ethylene glycol-modified pillar [5] arene (EGP5) and ATO [28]. Owing to the thermo-responsiveness of EGP5 and plasmonic heating induced by the near-infrared absorption of ATO, the film exhibited an outstanding photo-thermochromic effect, with an excellent solar modulation ability (56.1%) and initial luminous transmittance (77.2%) (Figure 3d). The dynamics and reversibility of the host–guest interaction between EGP5 and the pyridinium unit can avoid the collapse and damage of the polymeric hydrogel structure, which solved the problem of its poor durability and the repeatability of the hydrogels. Then, Wu et al. [29] prepared Cs_x_WO_3_/PAM-PNIPAm smart windows, in which Cs_x_WO_3_ was the photothermal component, PNIPAm was the optical control switch and polyacrylamide (PAM) hydrogel was the skeleton of the hydrogel matrix, so as to prevent any damage to the hydrogel structure. This smart window system was mainly heated by the near-infrared(NIR) light. While maintaining a good visible light transmittance, nearly 96% of the near-infrared light can be shielded by this window, and the indoor temperature can be maintained at approximately 25 °C. It can greatly reduce energy consumption from sources such as air conditioning and heating.

Photothermal materials, such as GO and Cs_x_WO_3_, mainly use high absorption characteristics to convert light energy into heat energy. However, AuNRs and Cu_7_S_4_ used a local surface plasmon resonance effect to convert absorbed light energy into the kinetic energy of electron resonance, and then converted it into the vibration energy of the lattice through the lattice scattering of electrons [30]. The heat energy of lattice vibration was transmitted to the surrounding environment, thereby increasing the temperature of the environment. Cao et al. (Figure 3e,f) [31] prepared polyvinylalcohol/thermochromic dyes/AuNRs sunlight-responsive smart window films through combining the thermal discoloration property of the thermochromic material and the photothermal effect of AuNRs. Then, this composite material was combined with hydroxypropyl methylcellulose (HPMC) to prepare a smart window model with good optical properties. This model can achieve a stable optical performance transition under sunlight, and by controlling the content of AuNRs and the type of dye, a prototype of color smart windows with different switching temperatures can be achieved. (Figure 3g). Using a similar principle, Zhu et al. [32] prepared a smart window based on Cu_7_S_4_/PNIPAm hydrogel with a lower price and cost (Figure 3h). Based on the good light and heat effect of Cu_7_S_4_, this smart window can not only respond quickly at room temperature, but can also improve the indoor temperature in the cold.

### 2.3. Liquid Crystal Smart Windows

Liquid crystals respond to temperature stimulation by adjusting the orientation of anisotropic molecules. They show a high transparency at a low temperature and become blurred due to strong light scattering at a high temperature. They have broad application prospects in the field of smart windows. Through combining the liquid crystal polymer and the photothermal properties of Cs_x_WO_3_, a flexible multi-response smart film with excellent mechanical strength was obtained [33]. In actual application, the transmittance of visible light through temperature and infrared light can be controlled, and 95% of near-infrared radiation in the range of 800~2500 nm can be shielded by this film. This film can achieve a mass production by using a roll-to-roll process, which had great significance in the field of smart windows.

**Figure 4:**
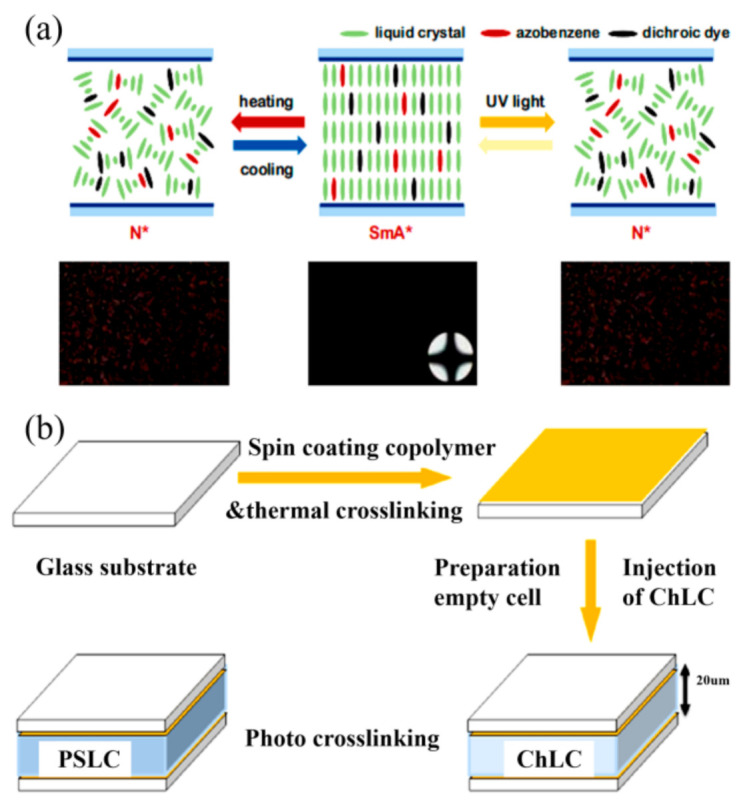
(**a**) Self-occlusion shutter schematic and POM images [36]. (**b**) Preparation process of polymer-stabilized liquid crystal (PSLC) smart window. Reproduced with permission from [37]. Copyright 2017, American Chemical Society.

In addition, liquid crystals are often combined with azo dyes to prepare UV-driven liquid crystal devices. Azo dye is an ultraviolet (UV) photochromic material that can change its molecular shape when irradiated by light. The trans-formed form azo derivatives is rod-shaped, which can stabilize the liquid crystal phase, whereas the cis form is curved, and when it exists, will destroy the stability of the liquid crystal phase [34]. Under UV irradiation, the directional alignment of liquid crystal molecules can be induced by the photoisomerization of azobenzene molecules, thereby driving its phase transition. Oh et al. [35] proposed a photoelectrically adjustable cholesteric liquid crystal doped with push-pull azobenzene. These liquid crystals can be stimulated by light or heat to achieve a reversible conversion between transparency and opacity. Then, Oh et al. [36] further doped push-pull azobenzene and chiral liquid crystals to prepare liquid crystal smart windows controlled by ultraviolet light and temperature. Under UV irradiation, the transformation of the liquid crystals, from the transparent chiral smectic phase (SmA* phase) to the opaque chiral phase sequence (N* phase), was induced by the trans-cis photoisomerization of push-pull azobenzene (Figure 4a). In addition, Kuang et al. [37] prepared a stable azobenzene copolymer brush, which was used as a substrate to prepare UV-driven polymer-stabilized liquid crystal (PSLC) smart windows (Figure 4b).

### 2.4. Chapter Summary

A recent progression of photo-driven thermochromic smart windows is introduced in this section. In order to achieve the transformation of the optical properties of thermochromic materials at room temperature, through using the photothermal conversion performance of photothermal materials, the surface temperature of the composite device was increased, and the optical performance of the composite device was changed. Research from single-band light-absorbing materials to multi-band light-absorbing materials has improved the optical performance of smart windows to the greatest extent.

At present, the research on photo-driven thermochromic smart windows is still in the preliminary stage, and its research is mainly concentrated on three types of VO_2_, hydrogels and liquid crystals. In the future, it is still necessary to explore the combination of different photothermal materials and thermotropic materials to further improve the optical performances and durability of smart windows, and to realize large-scale, industrialized production. So far, most of the research is still focused on the photothermal performance and optical performance of composite devices, whereas there is less research on the durability and mechanical performance of the composite device. This is an aspect that needs to be focused on.

## 3. Electro-Driven Thermochromic Smart Windows

Compared with the photo-driven thermochromic smart windows, the electro-driven thermochromic smart windows adds active regulation, which can independently adjust the transparency of the windows. The selection of electric heating materials and the structure of the electric heating layer were mainly researched in electro-driven thermochromic smart windows. The electrode materials with a low cost, mature preparation technology and high electrical and thermal conductivity tended to be selected. The structure of the electro-driven thermochromic smart windows was more complicated than those that were photo-driven, and its structure was also the focus of research. Further research is still needed in order to avoid the influence of the electric heating layer on the optical performance of the composite device and to increase the electric heating rate.

### 3.1. VO_2_-Based Smart Windows

**Figure 5:**
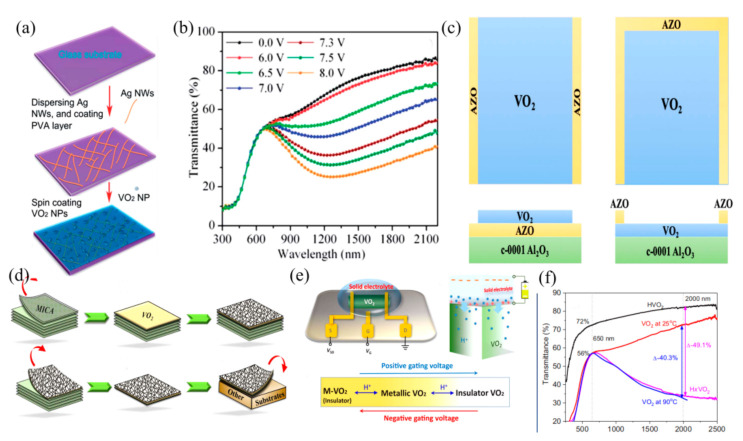
(**a**) The preparation process diagram of VO_2_NF/AgNWS electrochromic film on glass substrate. Reproduced with permission from [39]. Copyright 2014, Royal Society of Chemistry. (**b**) Transmission spectra of VO_2_NF/AgNWS electrochromic films under different applied voltages. Reproduced with permission from [39]. Copyright 2014, Royal Society of Chemistry. (**c**) VO_2_/AZO multilayer structure, AZO at the bottom and edge diagram. Reproduced with permission from [44]. Copyright 2020, Springer Nature. (**d**) CNT-VO_2_-MICA film preparation process schematic diagram. Reproduced with permission from [45]. Copyright 2017, Elsevier. (**e**) Gate control diagram of VO_2_ device with source, drain and gate. Reproduced with permission from [46]. Copyright 2019, The American Association for the Advancement of Science. (**f**) Optical transmission spectra of VO_2_, H_x_VO_2_ and HVO_2_ films. Reproduced with permission from [46]. Copyright 2019, The American Association for the Advancement of Science.

Due to the advantages of high transparency, high thermal conductivity and high electrical conductivity, electrothermal materials such as ITO and AgNWs have been integrated into different kinds of electro-driven thermochromic smart windows. Beydaghyan et al. [38] prepared VO_2_ films with a high transmittance and excellent thermochromic switching performance on the ITO layer. A VO_2_ phase transition was induced by Joule heating with ITO layer as the conductive layer. The transmittance of VO_2_/ITO films at 2500 nm has a reversible conversion from as high as 65% to near to zero. The particle size of VO_2_ deposited on ITO was smaller than that deposited on the glasses, which reduced the phase transition temperature of VO_2_ to a certain extent. Compared with the heating plate, the phase transition temperature of VO_2_ deposited on ITO is lowered by 4~7 °C. On this basis, Li et al. [39,40] studied the optical properties of VO_2_-based electro-driven thermochromic smart windows with AgNWs and ITO as conductive materials. The decrease in the infrared spectrum can be observed in the AgNWs/VO_2_ device at the voltage of 6.5–8 V (Figure 5a,b), and a further increasing of the voltage no longer leads to a decrease in the infrared spectrum. The same phenomenon was produced in ITO/VO_2_ devices at approximately 12.5–15 V. However, when the voltage was applied, the ITO substrate had a strong absorption rate at 2500 nm, and its infrared switching performance could be extended to near infrared regions.

In practical applications, how to reduce production costs and realize large-area heating is a problem to be considered in electric-driven thermochromic smart windows. The preparation technology of ITO has become mature, but the price is expensive. Therefore, using ITO as an electric heating device will greatly increase the preparation cost. Generally, the use of cheap and stable electrodes, such as fluorine-doped SnO_2_ (FTO) [41,42], Al: ZnO (AZO) [43,44], will be more conducive to the development of electrically driven thermochromic smart windows.

Both FTO and ATO were transparent conductive oxides that were easy to prepare and had a low cost. The optical constant of FTO is between VO_2_ and glass, and the dielectric constant is similar to VO_2_. Therefore, the optical performance of VO_2_ can be effectively improved by introducing an FTO buffer layer between glass and VO_2_. It has been proven that that VO_2_ phase transition can be driven by applied voltage on FTO/VO_2_/FTO multilayer films [38]. However, the threshold voltage also increased with the increase in the area of FTO and VO_2_ [41,42]. Then, Xu et al. [42] directly deposited VO_2_ on FTO glass. The experiment results showed that the rapid phase transition of VO_2_ in a large area can be achieved by applying a voltage below 6 V.

Similarly, studies have shown that, as long as a weak electric field is applied to the AZO/VO_2_ film, the fine control of the phase transition of the VO_2_ film can also be realized [43]. Furthermore, the structure of the AZO/VO_2_ film is also the focus of research. A suitable structure can reduce the voltage demand and further reduce energy consumption. The optical properties of AZO/VO_2_ multilayer films with different structures were studied [44] (Figure 5c). The results showed that depositing AZO on the edge of VO_2_ can maintain the Joule heating effect and the phase transition amplitude. The phase transition temperature of VO_2_ was reduced by introducing strain at the same time. Compared with depositing VO_2_ on AZO, this structure was more suitable for electric-driven thermochromic smart windows.

In addition to using the Joule heating effect to drive the phase transition of thermochromic materials, the phase transition can also be regulated by directly using bias voltage. In order to overcome the limitation of the high phase transition temperature of VO_2_, Chen et al. [45] adopted the method of directly growing VO_2_ on layered mica sheets and integrating it with highly transparent single wall carbon nanotube (SWNT) films (Figure 5d). By adjusting the bias current, it is possible to change the starting local temperature and shift the initial situation close to the “phase transition boundary”, resulting in the decreased energy barrier in order to trigger the MIT behavior. This device has important application prospects in the future. Further, Chen et al. [46] modulated the reversible form of H from the VO_2_ lattice with a solid electrolyte layer assisting gating treatment. The insulation–metal–insulation tristate phase transitions of VO_2_ were realized (Figure 5e). The transition between H_x_VO_2_ and HVO_2_ can produce an obvious electrochromic effect at room temperature. The dramatic increase in the visible/infrared transmittance due to the phase transition from the metallic (lightly H-doped) to the insulating (heavily H-doped) phase results in an increased solar energy regulation ability of up to 26.5%, while maintaining a 70.8% visible luminous transmittance (Figure 5f). This effectively overcame the defects of the traditional VO_2_ intelligent windows. In addition, Lee et al. [47] integrated electrochromic materials and thermochromic materials into a single device to achieve a single or dual response, but this device failed to achieve effective control of the VO_2_ phase transition. Therefore, Jia et al. [48] combined electrochromic and thermochromic materials to design an all-solid-state VO_2_-based multilayer device with a VO_2_/LiTaO_3_/WO_3_ sandwich structure. The reversible doping of Li in the VO_2_ lattice was controlled by the bias voltage. This not only avoids the degradation of the optical performance of VO_2_ caused by doping, but also effectively realizes the regulation of the phase transition temperature of VO_2_.

### 3.2. Hydrogel Smart Windows

Traditional hydrogels, such as PNIPAm, are easily dehydrated in harsh environments. Therefore, they have a poor thermal stability. At 60 °C, the total weight of PNIPAm decreased by 90% after 30 min due to the poor water retention ability [49]. In the photo-driven part, some researchers have already proposed solutions for this [28,29], and in the electro-driven part, Gyenes et al. [50] prepared an electrically driven polymer gel smart window. The polymer gel was composed of two thermally induced gel layers. The active hydrogel layer was used to adjust the optical performance of the window, and the inactive gel layer prevented the active gel from generating spatial separation after phase separation, which was used to improve the stability of the device. The optical properties of the gel layer can be controlled by changing the audio frequency alternating current. In this way, the electrolysis and gas generation of hydrogels can be avoided, and the durability of hydrogel smart windows can be further improved. Besides, Chen et al. [51] designed a thermo- and electro- dual response smart window system based on P(NIPAM-Dav) (diallyl-viologen) ionic liquid (IL) gel. The transmittance change in the fabricated devices was observed to be greater than 50% at a wavelength of 580 nm. After 20 continuous cycles, the transmittance had no obvious decay, indicating that the device had good stability.

**Figure 6:**
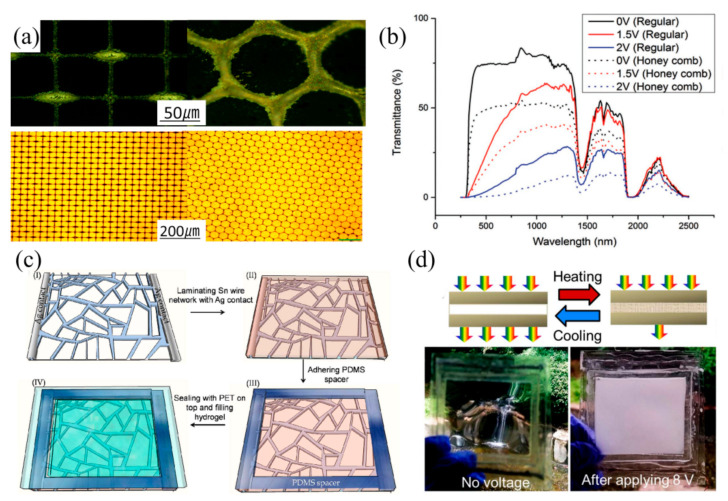
(**a**) Ag mesh electrode square and honeycomb arrays. Reproduced with permission from [52]. Copyright 2016, Wiley. (**b**) Sunlight transmittance of conventional and honeycomb arrays [52]. (**c**) Schematic diagram of the manufacturing steps of Sn/hydrogel devices. Reproduced with permission from [53]. Copyright 2017, Elsevier. (**d**) Cu/HPMC hydrogel before and after Joule heating [53]. Reproduced with permission from [53]. Copyright 2017, Royal Society of Chemistry.

Furthermore, in order to improve their optical performance and the response rate of electro-driven hydrogels smart windows, it is necessary to focus on the materials and structures of the electric heating layers [52,53,54,55,56,57]. Zhou et al. [52] prepared transparent conductive grids by using silver nanoparticles through a simple room temperature preparation process (Figure 6a) and studied the electrothermal effects of conventional grids and honeycomb structures. The results showed that the initial light transmittance of the honeycomb was 30% lower than that of the grid structure, but the heating efficiency was higher (Figure 6b). Then, in order to seek lower cost electrode materials, using Sn [53] and Cu [54] as conductive materials, a high connectivity metal wire mesh was prepared on the HPMC hydrogel layer by means of crack lithography. (Figure 6c,d). The Sn electrode had a light transmittance of approximately 80%. With the 8 V voltage applied, the transformation of the optical properties can be driven by the Joule heat generated by the Sn electrode. Similar electro-driven characteristics can be produced in the Cu electrode at a voltage of 4.5 V. Importantly, the variation of hydrogel optical properties can be driven by the two electrodes, with approximately only a 0.2 W/cm^2^ power consumption. They are inexpensive, and can be used as a substitute for electrodes such as Ag and ITO.

### 3.3. Liquid Crystal Smart Windows

**Figure 7:**
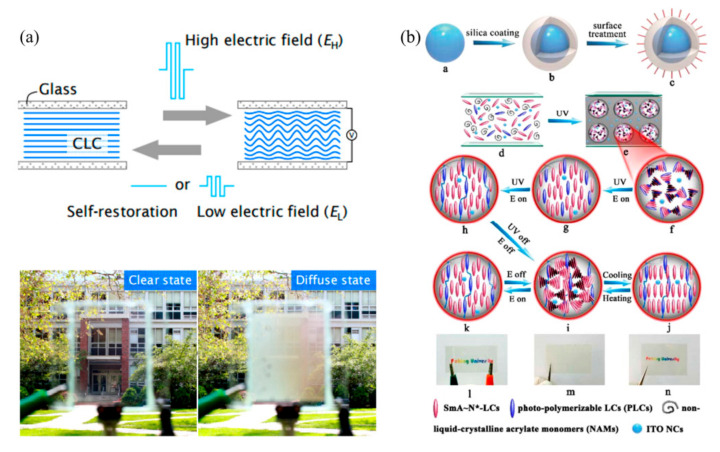
(**a**) Schematic diagram and photo of CLC smart window (d ≈ 45 μm). Reproduced with permission from [58]. Copyright 2018, Optica. (**b**) Schematic diagram of preparation and response of ITOncs/liquid crystal. (**a**–**c**): a single ITO NC is encapsulated with a hydrophilic silicon barrier to form a core/shell structure, and then subjected to methacryloylpropyl-trimethoxysilane (MPTMS) surface treatment; (**d**–**k**): Preparation procedures for the smart film: a homogenous polymeric syrup is sandwiched between two plastic transparent substrates (**d**), and the film is irradiated with ultraviolet light to form a porous polymer network and LCs droplets (**e**,**f**). Then, an electric field is applied to perpendicularly orient the LCs (**g**), meantime, a second step of UV polymerization is carry out to complete the cross-linking between PLCs in the LCs droplets to form orientated liquid-crystalline polymer networks in the porous structure (**h**). According to temperature or electric field, the original film (**l**) can be reversibly changed between transparent (**n**) and opaque (**m**). Reproduced with permission from [60]. Copyright 2017, Royal Society of Chemistry.

Cholesteric liquid crystals are also called spiral liquid crystals, and can selectively reflect the light incident along the spiral axis. Chen et al. [58] demonstrated a generally transparent smart window based on a cholesteric liquid crystal with negative dielectric anisotropy (Figure 7). The transparency of this window can be controlled through the field strength. When there was no voltage, it showed a well-arranged plane cholesteric texture, showing a transparent state. When applying voltage, it presented a diffuse state and became blurred.

In recent years, liquid crystal polymers have attracted extensive research interest due to their excellent physical and chemical properties. Liquid crystal polymers are mainly divided into polymer-dispersed liquid crystal (PDLC) and polymer stabilized liquid crystal (PSLC). PDLC has good mechanical properties, but, in the absence of electric field, its molecular arrangement is disordered and presents a fuzzy state. PSLC can stabilize the initial orientation of liquid crystal molecules through the network, showing a transparent state. Based on this, mesogenic functionalized graphene (MFG) is integrated with PSLC to prepare a film with a self-assembled chiral structure. The optical properties of this film can be controlled by electricity heat and near-infrared light [59]. Later, the good mechanical properties of PDLC were combined with the high initial transparency of PSLC to prepare a composite film of a phase-deposited polymer network [60], which had a good mechanical strength and processability. This film can regulate the visible light and near-infrared by responding to the stimulation of electricity, heat and ultraviolet light, has a region of 400–2500 nm and has excellent optical properties.

### 3.4. Chapter Summary

Compared with the photo-driven thermochromic smart window, the electro-driven thermochromic smart window added active regulation, which can independently adjust the transparency of the windows. The selection of electric heating materials and the structure of the electric heating layer were mainly researched in electro-driven thermochromic smart windows. Electrode materials with a low cost, mature preparation technology and high electrical and thermal conductivity tended to be selected. The structure of the electro-driven thermochromic smart windows was more complicated than that of the photo-driven; in order to avoid the influence of the electric heating layer on the optical performance of the composite device and to increase the electric heating rate, its structure was also the focus of research, and further research is still needed.

## 4. Conclusions

Aimed at the problem of thermochromic smart windows responding to high temperatures in practical applications, this paper summarized the research progress of photo-/electro-driven thermochromic smart windows. It is generally accepted that photothermal materials or electrothermal materials were used to cooperate with thermochromic materials to control the incidence of sunlight at room temperature. This method can effectively solve the problem of an excessively high response temperature of thermochromic smart windows, further increasing the application range of thermochromic smart windows. This can promote the development of smart windows in terms of no energy consumption and a high popularity. However, the current photo-/electric-driven thermochromic smart windows are still in the preliminary stage of development. To commercialize the photo-/electric-driven smart windows as soon as possible, future research should focus on the following three aspects.

(1) Photo-/electro-driven thermochromic smart windows were still limited by the characteristics of the materials, and each material used in reports now has certain shortcomings. In the future, it is still necessary to modify these materials and develop novel photothermal materials, and it is urgent to explore the combination of different photothermal materials and thermotropic materials in order to improve the optical performance of smart windows;

(2) To promote the commercialization of photo-/electro-driven thermochromic smart windows, smart windows that have good mechanical effects, a low cost, are easy to promote and have long-term stability need to be prepared. Therefore, reducing the cost of materials and improving the stability and mechanical properties of devices are crucial in promoting commercial production;

(3) Photo-/electro-driven thermochromic smart windows have greatly promoted the development of smart windows in the direction of no energy consumption. The next development trend is to combine them with energy storage devices to collect energy and further promote energy conservation.

## Data Availability

Not applicable.
